# Report of the Council of the Epidemiological Society

**Published:** 1855-12

**Authors:** 


### REPORT OF THE COUNCIL OF THE EPIDEMIOLOGICAL SOCIETY.

April, 1855.

THE Council beg to present to the Society the following Report of its progress during the last twelve months, and of its present condition and prospects.

In the course of the year six Resident Members, four Non-resident Members, one Honorary Member, and six Corresponding Members have been added to the Society; four Resident Members have withdrawn. The Society has also to deplore the loss, by death, of a Vice-President, two Resident Members, and one Corresponding Member.

The following are the present numbers of Resident, Non-resident, Corresponding, and Honorary Members:—

| | |
|---|---|
| Resident Members . . . . | 100 |
| Non-resident ditto . . . . | 27 |
| Corresponding ditto . . . . | 78 |
| Honorary ditto . . . . | 6 |

When the last Council Report was presented, allusion was made to the steps taken by the Council, to add to the funds of the Society, so as to enable them to carry on with efficiency the inquiries of the various Committees and other operations of the Society.

The result of an application by the Council to the Government for a pecuniary grant was then pending, and hopes were held out that the result would be favourable. The Council, with the view of strengthening their application, addressed on the 14th September last a letter to the Right Hon. Viscount Palmerston, then Secretary of State for the Home Department, setting forth the objects and condition of the Society, and suggesting that his lordship might see fit to use his influence in forwarding the claims of the Society upon the Government.

The Council have sufficient reason to be aware of the interest Lord Palmerston takes in the Society; but they are obliged to add that, by a letter received from his lordship in December last, they learned with deep regret that their application, as well as that of his lordship to the Lords of the Treasury, for the desired assistance, had not been successful. Their lordships, in their letter dated November 24th, 1854, stated, that "they had found it to be necessary, as a matter of principle, to decline making grants of money for the use of voluntary Societies, however useful may be the objects which they have in view." Their lordships therefore expressed "their regret that, as the present case presents no peculiar feature to make it an exception to the rule which they have been obliged to lay down, they cannot comply with the request of the Epidemiological Society."

In these circumstances, the Council have felt it their duty to take into consideration the propriety of making an appeal to the public, in accordance with the proposition of Sir Charles Mansfield Clarke. They feel as strongly as ever, that a Society like this, whose labours are devoted to objects of public utility, is entitled to public support. And, as a preliminary step to further measures, they have resolved to distribute among the profession and the general public, a short statement explanatory of the objects, proceedings, and results of the Society, with the view of making them generally known, and of enlarging the sphere of the Society's usefulness.

The following papers have been read at the ordinary meetings, during the last twelve months:—

Monday, April 3rd, 1854. "Importance of providing the labouring classes with nurses in the time of epidemics, and other sickness; and a scheme by which it may be accomplished." By E. H. Sieveking, M.D. Read by the Author.

Monday, May 1st. "An account of the Indian plague, which prevails in the mountainous districts of Gurhwal and Kumaon; abridged from the official reports of the disease; with remarks on the analogy of its morbid phenomena, and mode of propagation to those of true plague." By James Bird, M.D. Read by the Author.

Monday, June 5th. "Plague and yellow-fever, in relation to quarantine." By Sir Wm. Pym, K.C.H., Superintendent-General of Quarantine. Read by Dr. McWilliam.

Monday, July 3rd. "The use of vegetable and mineral acids, in the treatment, preventive and remedial, of cholera, and other epidemic disorders of the bowels." By J. H. Tucker, Esq. Read by the Author.

Monday, August 7th. "Practical results of quarantine." By T. Spencer Wells, Esq. Read by the Author.

Monday, November 6th. An introductory address was delivered by the President of the Society.

Monday, December 4th. "The salubrity of Birmingham, its suburbs, and neighbourhood; and particularly the comparative immunity of that town from Asiatic cholera, and other epidemic diseases." By George Bodington, Esq., of Sutton Coldfield. Read by Dr. McWilliam.

Monday, February 5th, 1855. "The cholera in Tynemouth, in 1831–32, 1848–49, and 1853." By Headlam Greenhow, M.D. Read by the Author.

Monday, March 5th. "On the importance of recording the progress of epidemics, and on the best means of accomplishing this object in England and Wales." By B.W. Richardson, M.D. Read by the Author.

The Foreign and Colonial Secretaries continue to receive, from time to time, communications of value from their respective correspondents.

Dr. Milroy, Secretary for the West Indies and North America, has received a considerable amount of information regarding the severe visitations of cholera in Barbadoes, Grenada, and other West India islands which suffered from the pestilence in the course of last year. Dr. Bowerbank, of Jamaica, has sent to the Society a copy of the "Notification of the Central Board of Health of Jamaica," of which he is a member, on cholera,—a document which has been so highly approved of by the Secretary of State for the Colonies, that he ordered it to be printed in this country, with the view of its being circulated in the British West Indian possessions.

Dr. Purple, our corresponding member in New York, continues to express the warmest interest in the welfare of the Society in the pages of the *New York Journal of Medicine,* of which he is the talented editor. He has recently transmitted to Dr. Milroy a number of valuable reports illustrative of the public health and the sanitary condition of New York and Boston; also copies of his interesting "Observations on some of the remedial Properties of 'Simaba Cedron,' and of its employment in Intermittent Fever."

The Council again desire to acknowledge their obligations to the press in general, but more especially to the professional press, for the publicity they have given to the proceedings of the Society.

The members of the Society are already aware of the establishment of a journal devoted to epidemiological and sanitary subjects, under the editorship of Dr. Richardson, a Member of Council. A feature in this journal of peculiar interest to the members of the Society is the dedication of a part of each number, paged separately, to such papers, reports, and proceedings, as the Council should think proper to publish as the authentic transactions of the Society.

Several valuable additions have been made to the library of the Society during the past year, for which the Council would here record their obligations to their respective donors. Among the books recently received may be noticed—

"Reports on Epidemic Cholera, drawn up at the desire of the Cholera Committee of the Royal College of Physicians. By William Baly, M.D., and William W. Gull, M.D., Members of the Committee." Presented by the College.

"Notification of the Central Board of Health of Jamaica." Presented by Dr. Bowerbank, through Dr. Milroy.

"Register of Deaths in Bombay during 1852." Presented by Sir James Cosmo Melville.

"Additional Experiments, proving the identity of Cow and Small-Pox." By John Badcock, Esq., Brighton. Presented by the Author.

"On the Mode of Communication of Cholera." By John Snow, M.D. Presented by the Author.

"On the Epidemic Mental Diseases of Children." By R. H. Cooke, Esq. Presented by the Author.

"Experiments on the Communicability of Cholera to the Lower Animals." By W. Lauder Lindsay, M.D. Presented by the Author.

"Report on cholera, which attacked the fleet on the Black Sea, in August, 1854; more particularly as relates to her Her Majesty's Ships, Britannia, Albion, and Trafalgar." Presented by Sir William Burnett.

"On Cholera in the Baltic Fleet, extracted from the medical returns, received during the summer and autumn of 1854." Presented by Sir William Burnett.

"A Report on the Sanitary Condition of Ramsey." By Dr. Waller Lewis. Presented by the Author.

"On the use of Vegetable and Mineral acids, in the treatment, preventive and remedial, of cholera, and other Epidemic Disorders of the Bowels." By J. H. Tucker, Esq.

The *Veterinarian*, the *Pharmaceutical Journal*, and the *Assurance Magazine*, continue to be supplied to the Society through the kindness of their respective editors.

*Small-Pox and Vaccination Committee.*—Your Council in the last Annual Statement alluded to the report, which had been presented by the Small-Pox and Vaccination Committee, on the subject of compulsory vaccination, and to the efforts which had been made by that Committee to procure the amendment of the Vaccination Extension Act, and its adoption by the legislature. It is now their gratifying duty, to be able to announce to you that the results of the first year's operation of that enactment have, notwithstanding the defects with which the bill is still encumbered, been of the most striking and beneficial kind; the public vaccinations of children under one year of age, in England and Wales, having increased from 201,271 in 1853, to 408,824 in 1854, or from 53 to 65 per cent. on all the births registered, But an analysis of the returns from which these numbers are derived, shews that even this result falls very far short of that which should be attained under a more proper system of administration, and justifies entirely the commentaries on the deficiencies in the legislative enactment. And in the system of administrative, which were made last session by the small-pox and vaccination committee. To expound and remove these deficiencies, and to introduce altogether a better system, are the objects to which this committee has, for some time past, been devoting its energies.

An elaborate exposition of the whole subject has been drawn up in the shape of a memorial to the President of the Board of Health; the main object of which is to prevent the transfer of the management of the public vaccinatories of the kingdom from the Poor-Law Board to the Board of Health, and to place it under competent medical superintendence. The Council have unanimously adopted this memorial, which has been forwarded by the President to the Minister of Health, and has since been moved for in the House of Commons. And they would take this opportunity of emphatically expressing their conviction, that until the suggestions therein contained, shall have been carried out, this country will not derive the full benefits of the magnificent discovery of Jenner. The Council dwell with great satisfaction on the labours of this Committee, and on the results which have so speedily followed them, as illustrations of the benefits expected to be derived from the foundation of the Epidemiological Society.

*Cholera Committee.*—Besides the first set of queries drawn up by the Cholera Committee, of which upwards of 1200 copies have been distributed at home and in the colonies, some hundreds of another and more brief series of questions were, at the suggestion of Lord Palmerston drawn up by the Committee, and forwarded through the kindness of the Lords of the Admiralty to the medical officers of the Baltic and Black Sea fleets.

The number of replies from the fleets has been very satisfactory; but the Committee regret to state, that the number of returns from the profession at home and in the colonies, has as yet fallen very short of affording a sufficient basis for a report on cholera. Within the last twelve months twenty-five copies of the original set of queries, and fifteen copies of the several papers that detail the origin, objects, and progress of the Society, have been forwarded to each of the Indian Presidencies, through the Honourable the Court of Directors, with a view to their distribution among the medical staff of the Indian army. No reply to these communications has as yet been received; though the Council cannot doubt that the wide field which India presents for interesting research into the medical economy of troops, and into the sanitary condition and diseases both of prisoners in jails, and of the civil population at large, will not be left uncultivated; and that their medical brethren in that part of the globe will be ready to favour the Society with their co-operation in carrying out their investigation into cholera, and in other objects of epidemiological inquiry.

*Epizootic Committee.*—The Council confidently look forward to the early presentation of the Report from this Committee; and they feel assured that such a document cannot fail to be interesting and useful to all agriculturists and cattle proprietors, as well as to members of the medical and veterinary professions.

*Hospitals' Committee.*—This Committee have now in hand a considerable number of replies to their queries, from foreign as well as from home hospitals; but, from the extent and peculiar nature of the inquiry entrusted to them, they are unwilling to commit themselves to any positive promise as to the time when their Report will be ready.

*Committee on Supplying the Labouring Classes with Nurses in time of epidemic and other sickness.*—Since the last annual meeting, another committee has been appointed by the Council, which promises, if at all adequately supported, to form an important element in the social economy of the poorer classes of our labouring population. This Committee has for its object the consideration of the question of supplying the labouring classes with nurses in epidemic and other diseases Since the formation of the committee (May 12th, 1854) they have held numerous meetings, and have continued actively engaged in the prosecution of the object for which they were appointed. An address was issued very soon after the committee was instituted, in which their views, arguments, and general plans, were briefly stated. The following is a copy of the address:—

"*Committee for supplying the Labouring Classes with Nurses in time of Epidemic and other Sickness.*

"Dr. Sibson, *Chairman;* Mr. Aldrich, Dr. Camps, *Treasurers;* Dr. Burford Carlill, Dr. Hall Davis, H. T. Davies, Esq.; Dr. Hare, W. Filliter, Esq.; R. D. Grainger, Esq.; I. N. Jakins, Esq.; Dr. M'William, Dr. Snow, and J. H. Tucker, Esq.

"A Committee has been appointed by the Epidemiological Society for the purpose of determining the feasibility of a plan to secure throughout the country a staff of nurses available for the labouring population, when attacked by epidemic disorders, such as fever, cholera, or the like, when at any time overtaken by sickness, or during the period of child-birth. The acknowledged want of nurses, in addition to proper medical advice, does not require to be dwelt upon; there is no difference of opinion in the medical profession as to the great advantages to be derived, in a medical point of view, from the assistance of a well-skilled nurse, whether among the wealthy or the poorer orders of society. But the Committee are of opinion that there are other grounds than those of the speedy recovery of the individual patient, which call for the serious attention of the community at large to the proposition, and render it not only the duty, but the interest of society to acknowledge and provide for this want.

"Sickness weighs upon the poor much more heavily than it does upon the affluent, inasmuch as it affects their means of acquiring a livelihood, or diminishes to a disproportionate extent their daily earnings. It is, in innumerable instances, a source of ruin and degradation to families, who are consequently thrown upon the parish for relief, while it abridges not only their happiness, but also their life, and thus, directly or indirectly, diminishes the wealth of the country.

"The tax that is laid upon every member of a working family in health is a sufficient burden, and any addition in the shape of attendance upon the sick, whether the father, the mother, or a child, is too commonly more than can be borne with impunity. Sickness, from the want of proper and early precaution and separation, spreads more easily among the poor than among the rich. The spread of disease among the labouring classes affects the wealthy classes in a variety of ways; by the extension of disease, the loss of service, the indirect cost in charitable institutions, and the direct cost in increased poor-rates. It must be self-evident that anything incapacitating the father of a family from work, vitally and immediately affects the well-being of the whole household; it is scarcely less so when other members are afflicted. For example, the wife of a labouring man is taken ill, and has no assistance; she therefore loses not only the power of attending to her young family and domestic duties, but probably, also, some little earnings as a charwoman or sempstress; the husband returns from his work to a comfortless home, and is required to act as nurse to the patient and to the children; he is taxed beyond his strength, and falls ill in consequence; both parents succumb, and the offspring becomes the charge of their parish. Or, again, a widow with a small family is called upon, in the case of illness of a child, to choose the alternative of sacrificing the pittance she can earn by remaining at home, or of leaving her sick child alone, or perhaps to the careless supervision of an elder child or a neighbour. Results equally distressing as detailed in the former instance take place here; such and similar instances are of daily occurrence; we all cannot but see and feel the final issue in more or less glaring colours, but we fail to recognise one very important link in the chain of causes leading to such results.

"It is manifestly impossible to enter into a minute history of disease in its effects upon the labouring classes, but the foregoing sketches may serve to illustrate the politico-economical view upon which the Committee base their opinion; that in a pecuniary, as well as a sanitary, respect the community would be materially benefitted by supplying the assistance to the family of the labouring man, *which a nursing attendant, a person who would superintend the wants of the sick while she assisted in the domestic economy, alone could afford.* It is desirable that such person should be of the best moral and physical constitution; but it is not desirable that she should be of a class, or possessed of habits distinct from those of the parties to whom she would be sent. It would be as much her duty to adapt herself to the narrow circumstances of the case, and make herself generally useful, as to pay attention to the sick person. The nurse obtained from the workhouse, from her circumstances and previous habits, would be especially calculated to appreciate the necessities, and to aid in the domestic arrangements of the household to which she would be sent. She would thus diminish the burden borne by the healthy, and enable them to continue in their daily avocations; she would materially aid in the speedy recovery of the sick, and take off some of the harassing reflections and anxiety which now inordinately weigh upon the invalid, and cause him or her to return to the *ordinary duties of life long before the physical powers are properly restored.*

"The Committee are strongly of opinion that, if it were possible to provide a staff of nurses easily available throughout the country, and at given points, a material benefit would accrue, perceptible not only by the shorter duration of disease, and the consequent diminution of mortality and pauperism among the labouring classes, but also by the more complete and more speedy arrest of contagious disorders, which spread to all classes, and by the diminution of poor- and county-rates, which are, in a great measure, levied to provide against the direct or indirect effects of suffering, preventible by the assistance contemplated. The Committee hold that the sanitary measures which mark the progress of modern civilization are of vital importance; but they are also of opinion, that under the most efficient sanitary system, there will yet always remain a large amount of sickness claiming the aid of the medical attendant and the nurse. While they regard the want as a national want, they are convinced that the remedy should be a national one. And they consider that the scheme which they propose for adoption will render it possible to meet the requirements of the case, while it will, at the same time, with a little labour, confer a great boon upon a class of persons who, at present, are too often placed almost beyond the reach of hope.

"It appears from the Fourth Annual Report of the Poor-law Board, that in each of the 553 Unions in England there are, on an average, twenty-three able-bodied females, and fifteen able-bodied men. It is proposed that it should be made imperative upon the guardians of the poor in every parish to select from these able-bodied paupers whose moral conduct and general aptitude shall best qualify them for places of trust, and to give them the opportunities which the workhouse infirmary at once affords, of acquiring some knowledge of the management of the sick. It is proposed that these persons should be sent out as nurses gratuitously to all such sick persons in the Union as present a certificate of illness from the union medical officer, and that a small fee should be taken from all members of the labouring classes who make application to the workhouse for a nurse, when supported by a certificate signed by any other medical man but the Union officer. In some instances the nurse might return to the workhouse at night, partly because her services would then not be so urgently required, owing to the presence of all the members of the family; partly because the poor man's dwelling would not suffice for her accommodation. All monies paid for the nurse should go to the workhouse authorities, who should employ it for the establishment of premiums to the best behaved, by which means an inducement to good conduct would be held out.

"The clergy of the parish, the medical men of the neighbourhood, the visiting societies may not unreasonably be expected willingly to co-operate in exercising the necessary supervision, and thus aiding the workhouse authorities. Should the plan work so well as gradually to render many of the nurses independent of workhouse support, they might still be kept on the parish staff, as a list of nurses willing to attend at the fixed fee, and under the general supervision alluded to. As soon as the sense of pauperism is removed by the independent exertion of the individual, his *morale* is raised, and the community becomes doubly a gainer; while the good opinion of the public once secured in favour of those workhouse inmates who were selected to serve as nurses, would in itself act as an incitement, probably even stronger than that of a pecuniary reward.

"The Committee are unable in a brief statement, as the present must necessarily be, to enter more minutely into a scheme, affecting so important and extensive a system as that of the workhouses of England. It is their desire to draw public attention to the subject; they ask for co-operation for no small purpose, nor with any selfish view: they desire to fulfil a duty, imperative upon all who are in a position to cope with a defect of magnitude in the social system; with a defect, which no private charity alone can remove, and which, in this country, can never be adequately met by an ecclesiastical institution. Moreover, the Committee are of opinion that while by their scheme they immediately propose to supply nurses to the lower orders, they may anticipate that when public opinion is earnestly directed to the subject, the position of the nurse will become more and more honourable, as it is, undoubtedly, one of the holiest spheres of Christian, and more particularly female charity; females generally will come to regard the office of nurse as one of dignity, and a superior class will gradually be found to enter the ranks; but while the Committee confidently hope for these and other results from the agitation of the question, and more particularly from the adoption of some such scheme as the one suggested, they wish to confine themselves at present to the immediate practical solution of the main question.

"They sum up their argument in the following propositions: a system of nurses for the labouring classes may be regarded as a material aid in the cure and prevention of disease, and of its consequences, destitution and pauperism. The workhouses afford the *nuclei* existing throughout the country, in which the system may be initiated by employing the able-bodied women and men, now a source of unproductive expense to the country; every case of illness and death prevented is a pecuniary saving to the community, while it is a direct diminution of the amount of misery and suffering entailed upon the poor; the question is, therefore, one involving not only the interests of one class of society, but of every member of the community, whether high or low.

"RICHARD HOLT, M.A.

"EDWD. H. SIEVEKING, M.D. } *Hon. Secretaries.*

"The Committee of the Epidemiological Society appointed to prosecute this matter are entirely left to their own resources; it is, therefore, absolutely necessary that they should appeal to the friends of the undertaking for pecuniary aid, without which it will be impossible to meet the expenses incident upon their labours; any of the members of the Committee will gladly receive *contributions* for the purpose, or afford information on the subject."

---

The address was forwarded to the Poor Law Board, as the proposition by which the supply of nurses was to be secured throughout the kingdom essentially affected the internal administration of the workhouses. The President of the Poor Law Board received a deputation of the Committee, which was introduced by Dr. Babington, on the 24th of June, and, having listened to the statements of the members, he expressed his opinion that the plan, as it then stood, could not be carried into effect by the Board without further powers, which could only be conferred by Parliament. The Committee, with a view to obtaining further evidence on the importance, in a sanitary and politico-economical point of view, of supplying the lower orders with nurses, issued a series of queries to the medical officers of every Union of the kingdom. The general tenor of the answers received was strongly in favour of the objects pursued by the Committee. The Committee sought to enlist the assistance of a Medical Relief Committee of the House of Commons, then sitting; but that committee being on the eve of dissolution when the application was made, no aid could be afforded. An application to the President of the Board of Health promised a more favourable result, as the deputation of the Committee were requested to submit a statement which might be laid before the medical council of the Board, whose report the president engaged to introduce in the report which he would make to Parliament. The statement was forwarded, but the Committee have not since ascertained any definite result. In the meantime, from inquiries made, and the further experience required, it was concluded that a certain modification of the original plan would materially facilitate its adoption; and, in consequence, the following proposition was, after very careful and matured deliberation adopted, as the one to be finally promulgated by the Committee.

"A Proposition for qualifying Female Inmates of Workhouses, to act as Nurses in Epidemic or other Sickness.

"The Committee have ascertained that the workhouses of England and Wales, contain a large number of females who might be qualified to act as nurses,* and that the workhouse affords the means of training them, of testing their efficiency, and of rendering them generally available.

"In order to effect this it is proposed:—

"I. That the master and matron of every workhouse shall give such female inmates a routine of occupation, that shall afford them a knowledge of the duties required in the management of the sick.

"To carry out the plan proposed, it would be requisite that a General Order of the Poor Law Board be issued to every Board of Guardians, directing this and the following provisions to be enforced in their respective workhouses. This plan would entail no organic change in the classification and management of the inmates. Each female on being admitted would be put to the employment for which she appeared best fitted. After receiving the necessary instruction in the culinary and domestic department, she would be transferred to the infirmary; where, under the superintendence of the matron, nurse, and medical officer, she would be able to acquire a proper knowledge of the duties of nurse. Many of those inmates classified as "Aged," might be instructed in the same way. Even those who might leave the house before being fully qualified as nurses, as well as those who, having acquired the full qualification, did not select nursing as an occupation, would be materially benefited by the training, and rendered more useful to the society to which they might afterwards belong.

"II. That the medical officer of each workhouse, as soon as he shall consider an inmate competent to undertake the nursing of the sick out of the workhouse, shall certify to that effect.

"The master and matron of the workhouse would regulate the matters of detail with regard to the earlier stages of the training, and judge of those whose behaviour, character, and general aptitude would qualify them to be trusted to attend upon the sick. The medical officer, alone, would determine the character of the certificate, and the period at which the certificate should be awarded.

"III. That a register shall be kept at the workhouse of all those who have been certified by the medical officer as qualified nurses, containing their names, ages, certificates, and addresses. This register shall be open to the medical profession, the clergy, and the public at large, as a ready means of obtaining a nurse suitable to their wants.

"The registers would, collectively, form a source from which nurses might be selected, not only for private individuals, but also for

---

* According to a return moved for by Mr. Sotheron, in the House of Commons in 1854, there are no less than 13,352 able-bodied women in the workhouses of England, of whom 5,634 are of good character.

public institutions, and so embody a staff of nurses easily accessible throughout the kingdom. A means of livelihood would thus be opened to the workhouse inmate, and her position would be raised. Society at large would receive a direct benefit, in regard to the cure and prevention of disease, an indirect benefit in the arrest of destitution and pauperism; whereby the parochial charges would be lessened, and an elevation of the average duration of life be effected."

The Council are gratified to state, that the Lord Advocate, in the course of last Session, succeeded in carrying through Parliament a Registration Bill for Scotland. They earnestly hope that the "Registration of Births and Deaths Bill" for Ireland, may soon receive the sanction of the Legislature.

Such is a brief exposition of the proceedings of the Society during the past year, and of its present condition.

If it be alleged that the results have fallen short of expectation, the Council would respectfully submit, that, while they can turn with great satisfaction to several instances of munificent individual liberality, they have not as yet met from the State, or from the general public, or even from the profession, that degree of support, without which, it is impossible that the investigations of committees, and the general operations of the Society, can be carried on with efficiency and vigour.

