# Erratum

**Published:** 1855-12

**Authors:** 


*Note.*—Through inadvertence, the press at p. 8 of the Transactions of the Epidemiological Society published in our first Number escaped correction: the reader is therefore requested to make the following alterations, ***which are very material*** ones, ***at once*** with the pen:

l. 27, for 53, read 33.
l. 33, substitute a comma for a full stop after the word 'enactment', and continue 'and in the system of administration'.
l. 5 from the bottom, for 'prevent', read 'procure'.
l. 4 from the bottom, for 'vaccinatories', read 'vaccinations'.

